# Influence Mechanism of PA on the Thermal Decomposition of RDX Based on ReaxFF MD and DFT

**DOI:** 10.3390/molecules31091549

**Published:** 2026-05-06

**Authors:** Siman Guan, Zhijun Wang, Jianping Yin, Ruijie Hao, Qing Ji

**Affiliations:** 1 School of Mechanical and Electrical Engineering, North University of China, Taiyuan 030051, China; 2 Northwest Institute of Mechanical and Electrical Engineering, Xianyang 712099, China

**Keywords:** RDX decomposition, PA-RDX, ReaxFF MD, DFT calculation, reaction mechanism

## Abstract

To elucidate the physicochemical mechanisms underlying the violent explosion triggered by nylon (PA) jet penetration into explosive reactive armor, the thermal decomposition behavior of RDX and the influence mechanism of PA on its thermal reaction were studied by reaction molecular dynamics simulation and quantum chemical calculation, which were compared with experimental research. The study reveals that the decomposition of RDX is primarily initiated through pathways such as N–NO_2_ homolysis, HONO elimination, and concerted ring-opening. The addition of PA reduces the energy barrier for N–N bond homolysis and provides hydrogen atoms to initiate HONO elimination via a heterogeneous pathway with a lower energy barrier, thereby promoting the initial decomposition of RDX. The free radicals produced by the decomposition of PA and RDX participate in a synergistic reaction, efficiently yielding stable products and significantly altering the distribution of intermediate species. The introduction of PA lowers the activation energy barrier for RDX decomposition and supplies hydrocarbon fragments as fuel for the reaction, facilitating rapid decomposition and initiation. This work clarifies the dual mechanism by which PA promotes RDX detonation from the perspective of microscopic reaction kinetics, providing theoretical insights for understanding and modulating the response of explosives under complex impact conditions.

## 1. Introduction

Explosive reactive armor (ERA) is a sandwich-charge structure mounted on the outer layer of main battle tanks. Its operational mechanism involves detonation upon impact by a metal jet, where the resulting flying plates and fragments disrupt and attenuate the jet, thereby reducing its penetration capability [1]. To counter this defensive mechanism, two primary damage modes have been developed: the “shock-initiation” mode based on metal jets or reactive jets [2,3,4,5] and the “penetration without detonation” mode employing polymer liners to form low-density jets [6,7,8]. Polymer jets can penetrate reactive armor without triggering its explosion, thereby avoiding disruption of jet integrity and stability by explosive fragments and rendering the ERA ineffective.

However, previous studies have shown [9] that under identical dimensional conditions, low-density jets formed from polytetrafluoroethylene (PTFE) (2.16 g/cm^3^) liners achieve penetration without detonation of ERA, whereas jets formed from nylon (PA) liners (1.14 g/cm^3^) detonate ERA during penetration. Notably, due to its lower density, the impact pressure at the head of a PA jet should theoretically be lower than that of a PTFE jet. Yet, despite this lower impact pressure, ERA is still detonated by PA jets. This suggests that the phenomenon cannot be explained solely from a mechanical perspective. Instead, it is necessary to examine the inherent material properties and further investigate the chemical reaction behavior between the liner material (PA) and the explosive under specific conditions in order to understand the interaction mechanism between the jet material and the explosive, thereby gaining deeper insight into the detonation mechanism of reactive armor. To ensure consistency and comparability in research, this study follows the experimental design of references [9,10] and selects cyclotrimethylenetrinitramine (RDX) as the explosive component for investigation.

Regarding the fundamental properties and decomposition mechanisms of RDX, scholars have employed various advanced experimental techniques—such as in situ Fourier transform infrared spectroscopy [11,12,13], mass spectrometry [14,15], and coupled thermal analysis methods [16]—to elucidate its complex thermal decomposition pathways. These studies have confirmed that homolysis of the N–N bond constitutes the initial decomposition step and have identified the primary decomposition products. Moreover, research has demonstrated that the particle size of RDX [17] and its combination with different additives—such as the desensitizer DOS [18], strong reducing agent LiAlH_4_ [19], and oxidizer AP [20]—can significantly alter its thermal decomposition behavior and kinetic parameters. Investigations into the application of RDX in energetic composite material design [21,22,23,24,25,26] have provided important theoretical foundations and experimental support for improving the safety performance, combustion characteristics, energy output, and synergistic effects among components in energetic systems. In previous work, we experimentally examined the influence of PA on the decomposition of RDX by means of ignition combustion and product characterization [10]; however, the underlying mechanisms and detailed reaction pathways between the materials remain insufficiently explored. The advancement of theoretical calculation and simulation methods offers a powerful tool for understanding the mechanism through which PA affects the thermal decomposition of RDX at the microscopic scale.

At present, theoretical computational research can generally be divided into two categories. The first primarily involves simulation methods based on reactive force field molecular dynamics (ReaxFF MD). This approach combines classical molecular dynamics with a dynamic bond-order description, enabling the simulation of chemical reactions without predefined pathways. It balances the accuracy of quantum chemistry with the efficiency of classical force fields, making it suitable for multiscale systems and widely applied in fields such as combustion, catalysis, and materials science [27,28,29,30]. In studies of the energetic material RDX, ReaxFF MD has been extensively used to reveal its decomposition mechanisms in condensed phases and complex environments. Molecular dynamics simulations indicate that the initial decomposition of RDX mainly involves cleavage of N–N bonds to form NO_2_ molecules, followed by hydrogen atom transfer reactions, yielding intermediates such as HONO, HO, and NO. Among the products, N_2_ and H_2_O are the most stable species, while NO_2_, NO, and HONO act as intermediates during the thermal decomposition of RDX [31,32,33,34,35]. Reactive molecular dynamics simulations are employed not only to investigate the elementary reactions of pure RDX [36] but also to study composite systems—such as interfacial interactions between RDX and other energetic components (e.g., AP, HMX [37,38,39] or with other materials [40,41,42]). Therefore, this work adopts the ReaxFF MD method to preliminarily explore the influence of PA introduction on the decomposition pathways of RDX.

The second category of computations is based on quantum chemical methods [35,43]. Calculations utilizing density functional theory (DFT) provide fundamental physicochemical properties of energetic materials at atomic and electronic scales, including equilibrium structures, vibrational frequencies, conformational rearrangements, etc. [44]. Quantum chemical studies have systematically compared three competing unimolecular decomposition pathways of RDX—HONO elimination, N–N homolytic cleavage, and concerted ring-opening—along with their energy barriers [43,45,46], and have elucidated autocatalytic cycles triggered by initial products [47], revealing early ring-opening mechanisms and key reaction channels. DFT methods are also employed in the design of modified RDX molecules [48] or in probing interaction mechanisms between RDX and other atoms/molecules [24]. Given the robust reliability and broad applicability of quantum chemical computational approaches, this study employs such methods to investigate the key reaction mechanisms between RDX and PA.

Previous studies have combined experimental, theoretical, and simulation approaches to advance the understanding of RDX thermal decomposition from macroscopic phenomena to microscopic kinetic processes. However, issues such as the competition and coupling of multiple reaction pathways in the condensed phase, as well as the interaction mechanisms between multi-component systems, still require further investigation. In particular, research on the reaction mechanism between PA and RDX, as examined in this work, remains relatively scarce. Understanding the physicochemical processes between these two is crucial for predicting the thermal decomposition behavior and detonation performance of RDX under polymer jet impact.

Building on earlier experimental work [10], this study systematically investigates the chemical reactions between PA and RDX and the influence of PA on the thermal decomposition of RDX by integrating ReaxFF MD simulations with DFT calculations. Firstly, ReaxFF MD simulations are employed to compare the reaction processes of pure RDX and the PA-RDX system at 3000 K, revealing the thermal decomposition pathways of RDX in the presence of PA and the interaction mechanisms between the two. Subsequently, based on the ReaxFF MD results, quantum chemical methods are applied to conduct a deep exploration of the initial reaction steps between RDX and PA and to elucidate the key mechanisms by which PA affects the thermal decomposition of RDX. This research aims to clarify the influence mechanism of PA on the thermal decomposition reaction of RDX, thereby providing a theoretical foundation for understanding its impact on the detonation performance of RDX.

## 2. Results

### 2.1. Analysis of ReaxFF MD Simulation Results

#### 2.1.1. Evolution of System Potential Energy

**Figure 1:**
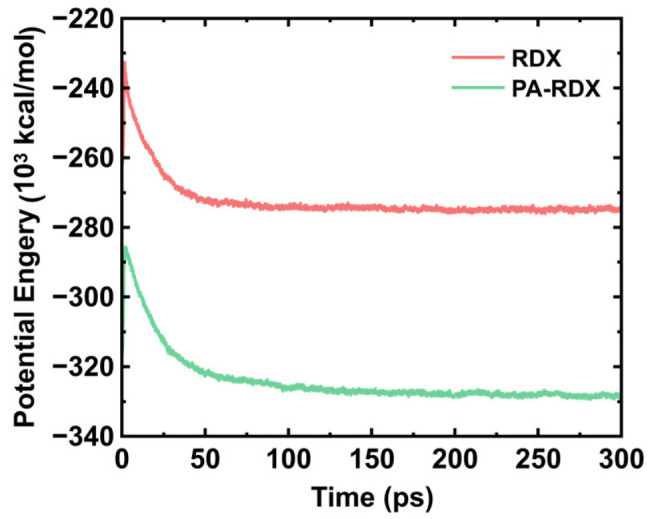
The potential energy change trend of the two systems at 3000 K.

The variation in potential energy over time for the two systems (after initial relaxation) at 3000 K is shown in Figure 1. During the thermal decomposition of explosives, the potential energy typically first increases, then decreases, and eventually stabilizes. This corresponds to three stages of chemical reactions: an initial rapid endothermic reaction stage, a chain reaction exothermic stage, and a stable product formation stage.

Taking the decomposition process of RDX as an example, the evolution in potential energy can be analyzed as follows: Explosive molecules are inherently in a metastable state. In the initial reaction stage (0–3 ps), the potential energy of the system increases. This is due to energy input from the external environment, which provides kinetic energy to the molecules and intensifies atomic vibrations. To initiate decomposition, the molecules must overcome an energy barrier, which corresponds to the activation energy required to break initial chemical bonds or rearrange molecules to reach the transition state. During this stage, the system absorbs heat from the surroundings, causing the potential energy to rise rapidly until it reaches its maximum. The decomposition reaction begins, and unstable chemical bonds break. Subsequently, the fragmented molecules and free radicals undergo further decomposition, generating gaseous products and solid residues. This process of bond breakage and new bond formation is accompanied by the conversion of chemical energy into thermal energy. Since the newly formed bonds are more stable and possess higher bond energy than the broken ones, the overall potential energy of the system decreases sharply. As the reaction gradually approaches equilibrium—thermodynamically stable low-potential-energy substances—final products are formed. During this stage, the potential energy of the system decreased slowly and finally stabilized, indicating that the reaction gradually reached completion.

#### 2.1.2. Product Analysis

To more clearly illustrate the origin and reaction pathways of various products in the system, ReacNetGenerator (v1.6.15) [29] was employed to analyze the major reaction routes. This method automatically extracts reaction networks from simulation trajectories and filters out trajectory noise using a hidden Markov model, thereby uncovering complex reaction mechanisms.

**Figure 2:**
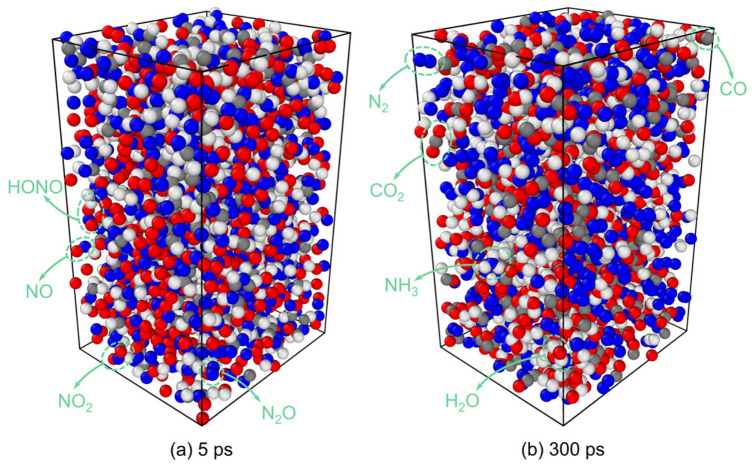
Snapshot of RDX decomposition for 3000 K.

**Figure 3:**
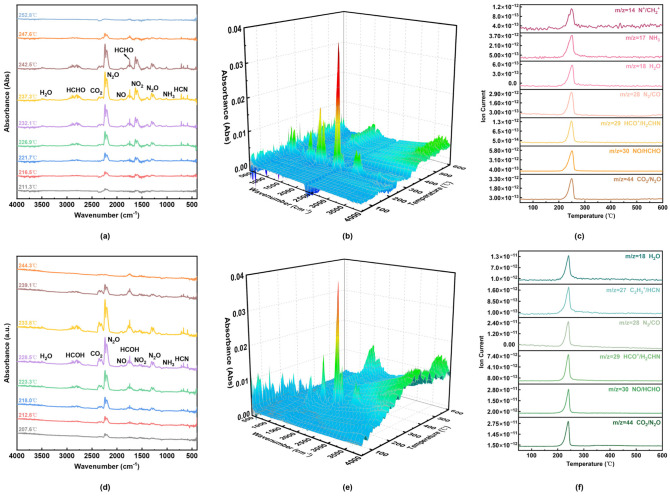
The infrared spectra, three-dimensional infrared spectra and mass spectra of the decomposition products of RDX and PA-RDX: (**a**–**c**) infrared spectra, three-dimensional infrared spectra and mass spectra of the decomposition products of RDX gas; (**d**–**f**) infrared spectra, three-dimensional infrared spectra and mass spectra of PA-RDX gas decomposition products [10].

Figure 2 shows snapshots of RDX decomposition at 5 ps and 300 ps obtained from ReaxFF MD simulations. The decomposition products include N_2_, H_2_O, CO_2_, N_2_O, NH_3_, NO_2_, NO, HONO, HCHO, HCN, and CO [19], which are in agreement with the gaseous products detected by the FTIR and MS experiments reported in Ref. [10] (Figure 3). This consistency confirms the reliability of the simulation results.

**Figure 4:**
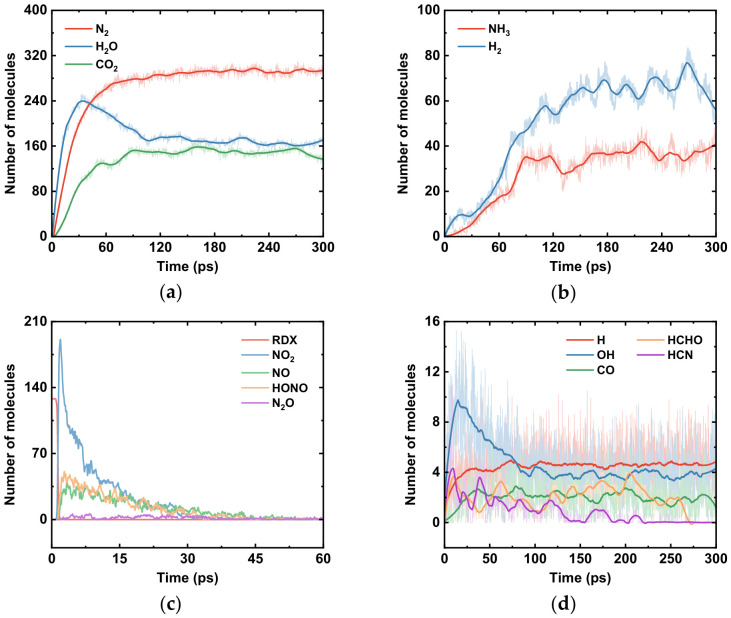
Changes in RDX thermal decomposition reactants and products: (**a**) main products; (**b**) intermediate products; (**c**) intermediate products; (**d**) intermediate products. (The thin semi-transparent line represents the raw data recorded at each time step; the thick solid line represents the curve smoothed using the Savitzky–Golay algorithm with 300 window data points).

**Figure 5:**
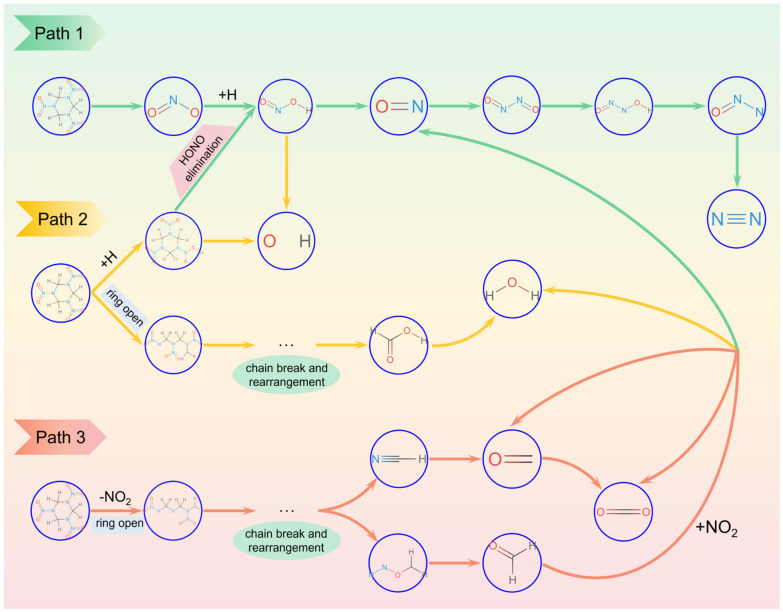
Main reaction paths in the thermal decomposition process of the RDX system.

Figure 4 shows the temporal evolution of various species in the RDX system, and the formation pathways of some major products (N_2_, H_2_O, CO_2_) obtained from ReacNetGenerator analysis are presented in Figure 5. When the reaction proceeds to 1.0 ps, the quantity of RDX molecules begins to decrease, while NO_2_ appears first among the products. This indicates that the N–NO_2_ bond is the first to break during RDX decomposition [49,50]. The initial step in fragment formation is often referred to as the primary reaction of thermal decomposition. The next small molecular groups to emerge are HONO and ·OH. These groups originate from the transfer of active hydrogen: an active H atom from the RDX molecule transfers to an O atom of the NO_2_ group, forming a HONO group. Subsequently, the HO–NO bond cleaves to yield an ·OH radical, which then participates in reactions leading to the formation of H_2_O molecules. Apart from intramolecular H transfer within RDX, NO_2_ radicals can also attack neighboring RDX molecules, abstracting a hydrogen atom from a methylene (–CH_2_–) group to generate HONO [46,47]. The HONO group further decomposes into ·NO and ·OH radicals. Through a series of chemical steps, ·NO ultimately contributes to N_2_ formation, while the ·OH radicals continue to produce H_2_O molecules.

The process described above constitutes a chemical chain reaction (also known as a radical chain reaction). A chemical chain reaction proceeds through highly reactive radicals as chain carriers, propagating via these radicals and undergoing numerous repetitive cyclic steps. The reactive NO_2_ group attacks RDX molecules, initiating the chain reaction. Newly generated radicals then propagate the reaction until stable molecules such as N_2_ and H_2_O are formed, at which point the chain terminates. Apart from the reaction pathways mentioned, H_2_O molecules can also be formed through a concerted ring-opening process, followed by a series of subsequent decomposition reactions. The decrease in the quantity of H_2_O molecules observed in Figure 4a results from secondary reactions (e.g., H_2_O + CO⇌CO_2_ + H_2_) and high-temperature decomposition effects. For instance, H_2_O can act as an oxidizer, reacting with solid carbon or incompletely oxidized hydrocarbon fragments to produce CO and H_2_ (Figure 4b), thereby being consumed itself. This leads to the observed trend in the RDX thermal decomposition simulation, where H_2_O concentration first increases, then decreases, and finally stabilizes (Figure 4a). Although H_2_O is inherently a stable product, it can still be converted through various pathways within the complex decomposition environment.

Following the elimination of NO_2_ groups from the RDX molecule, the molecule undergoes concerted ring-opening decomposition [31]. After a series of subsequent decomposition steps, HCN and HCHO (formaldehyde) are generated. HCN can further react to yield N_2_, while HCHO reacts with NO_2_ through the following pathway to produce CO_2_ and H_2_O:

5HCHO + 7NO_2_ → 7NO + 2CO_2_ + 3CO + 5H_2_O

As shown in Figure 4c, HONO is generated only during the early stages of the reaction and rapidly converts into stable products. Throughout the kinetic simulation, H atoms as well as ·OH and CO groups remain present in small amounts under dynamic equilibrium. Furthermore, it can be observed from Figure 4c that NO, NO_2_, and HONO groups appear only in the initial phase of thermal decomposition, while N_2_ is produced rapidly and abundantly during the same period. The final products of RDX thermal decomposition maintain a dynamic equilibrium in the later stages of the reaction. These findings are in good agreement with previous studies [51,52], supporting the reliability of the simulations performed in this work. Due to the complexity and scale of the radical chain reactions involved, it is not feasible to exhaustively list all possible reaction pathways. Therefore, the reaction pathway illustrated in Figure 5 represents only one route leading to the corresponding product; it is not the sole source of that product.

**Figure 6:**
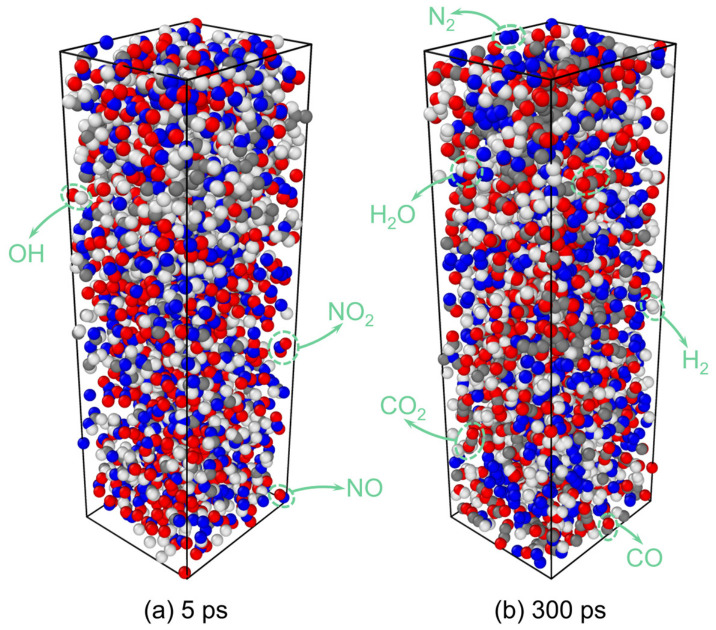
Snapshot of PA-RDX decomposition for 3000 K.

**Figure 7:**
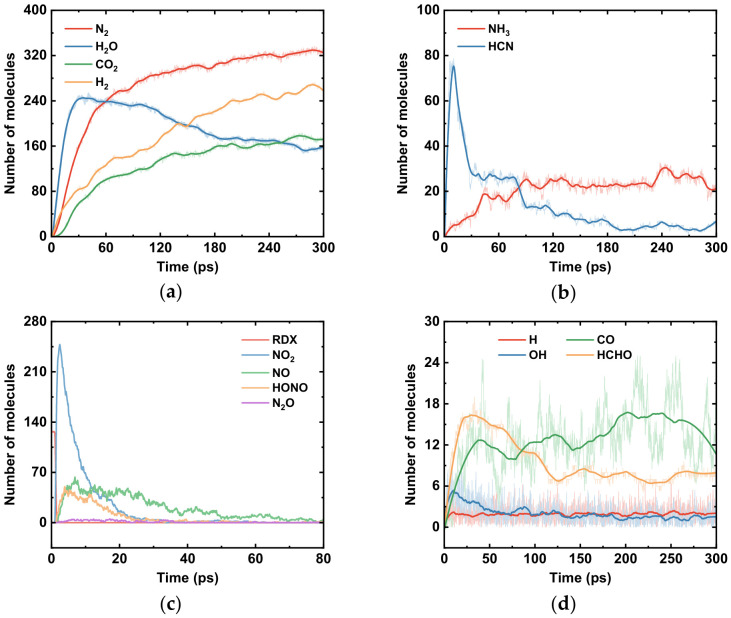
Changes in PA-RDX thermal decomposition reactants and products: (**a**) main products; (**b**) intermediate products; (**c**) intermediate products; (**d**) intermediate products. (The thin semi-transparent line represents the raw data recorded at each time step; the thick solid line represents the curve smoothed using the Savitzky–Golay algorithm with 300 window data points).

**Figure 8:**
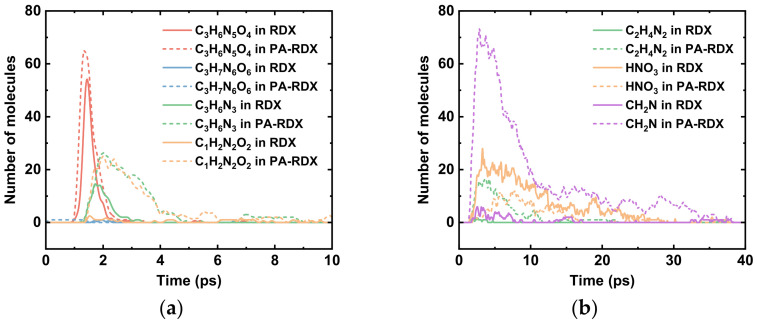
Changes in macromolecular fragments generated by the two systems: (**a**) macromolecular intermediate products; (**b**) other intermediate products.

Decomposition snapshots of the PA-RDX system at 5 ps and 300 ps are shown in Figure 6. By comprehensively analyzing the changes in reactants and major product species of the PA-RDX system (Figure 7), we find that the RDX molecules in this system first generate NO_2_ groups at the onset of the reaction. Comparing the evolution of the quantities of large molecular fragments generated in the RDX and PA-RDX systems over time, as shown in Figure 8, the first large molecule to appear in the PA-RDX system is C_3_H_6_N_5_O_4_, slightly earlier than its emergence in the pure RDX system. Moreover, the quantities of NO_2_ groups and C_3_H_6_N_3_ are much higher than those generated in the pure RDX system, indicating that the addition of PA promotes the homolysis of N–N bonds. Comparing the quantity of HONO groups in Figure 4c and Figure 7c, the PA-RDX system produces a slightly higher amount, suggesting that the addition of PA also facilitates the HONO elimination reaction. This inference is further supported by Figure 8a, which shows fewer C_3_H_5_N_5_O_4_ molecular fragments and more C_3_H_6_N_5_O_4_ molecular fragments in the PA-RDX system, implying that in the PA-RDX system, the H atom in the HONO elimination reaction is more likely to originate from PA molecules. We will further verify this viewpoint using DFT calculations. The addition of PA in the PA-RDX system introduces more hydrogen atoms, resulting in much higher H_2_ production compared to pure RDX decomposition. However, since the oxygen content in the system does not change significantly, it does not lead to increased H_2_O generation. Therefore, the difference in the quantity of H_2_O molecules between the decomposition products of the two systems is not notable.

The thermal decomposition of PA is primarily initiated by the cleavage of its amide bonds (–CO–NH–), following a free radical chain reaction mechanism. At high temperatures, PA molecules generate carbonyl or amino radicals, which attack amide bonds in the same or adjacent chain segments, producing new radicals that further induce chain scission. The cleavage process mainly involves random breaking of amide bonds (–NH–CO–) and methylene chains (–CH_2_–), generating smaller radicals such as hydrocarbon radicals (·CH_2_), oxygen-containing radicals (·CO, HCO·), and nitrogen-containing radicals (·NH) [53]. These radicals are relatively unstable and rapidly react to form more stable radicals, which further decompose into small gaseous molecules. Both PA and RDX molecules produce common secondary products, such as HCHO, HCN, CO, and NO, during pyrolysis [54], which explains why the quantities of these molecules in Figure 7 are significantly higher than those generated in the pure RDX system shown in Figure 4. The addition of PA introduces more nitrogen-containing active groups, promoting the autocatalytic reaction of RDX [46] and increasing the final yield of N_2_. In addition to the small gaseous molecules generated from PA’s own decomposition, the active radicals produced during its cleavage also interact with radicals from RDX decomposition, such as ·NH_2_ (PA) + ·NO (RDX) → N_2_ + H_2_O, similarly producing stable gas molecules. This enhances the overall gas production and accelerates RDX decomposition. Since oxygen-containing active radicals from RDX continuously oxidize hydrocarbon fragments generated by PA, forming HCHO, CO, and eventually CO_2_ and H_2_O, the quantity of CO_2_ molecules continues to rise until reaching a dynamic equilibrium after 180 ps.

Comparing the quantities of large molecular fragments generated in the two systems shown in Figure 8, we observe that the PA-RDX system produces a richer variety of RDX-derived large molecular fragments during decomposition than the pure RDX system. This phenomenon indicates that the presence of PA enables more RDX molecules to rapidly depart from their initial intact structures, thereby initiating the decomposition process. The presence of PA reduces the energy barrier for RDX decomposition, which will be elaborated on in Section 2.2, allowing more molecules to undergo incomplete decomposition earlier, suggesting that PA facilitates the early-stage decomposition of RDX and promotes the transformation of RDX from “intact molecules” to “fragments”. Additionally, the quantity of CH_2_NNO_2_ groups generated via concerted ring-opening of RDX in the PA-RDX system is significantly higher than that in the pure RDX system. Consequently, the further decomposition products of this group, such as HCN, are also produced in higher yields in the PA-RDX system. The addition of PA introduces more molecular fragments, increasing the probability of collisions and recombination among fragments. This accelerates the transfer and proliferation of reactive sites, thereby macroscopically enhancing the overall extent and rate of decomposition [10].

To evaluate the reliability of the simulations, we investigated the decomposition kinetics of each system at high temperatures ranging from 1000 K to 3000 K. Extensive previous studies have thoroughly described the use of reactant consumption rates to analyze first-order pyrolysis kinetics [55,56]. The kinetic model adopted in those works assumes complete conversion of the reactant into products. In this study, the concentration of the reactant was simply represented by the number of molecules. The rate constant *k* at each constant temperature *T* was then determined by linearly fitting the natural logarithm of the number of molecules *N_t_* against simulation time *t*, as shown in Equation (1), where *N*_0_ denotes the initial number of molecules—in this case, *N*_0_ = 128 for RDX.

(1) $$\ln N_{t}-\ln N_{0}=kt$$

The Napierian logarithm of the rate constant *k* (i.e., ln *k*) was then plotted against the inverse of the absolute temperature *T* (i.e., 1/*T*) based on the Arrhenius expression given in Equation (2), allowing the calculation of the activation energy *E_a_* and the pre-exponential factor *A*. Here, *R* represents the molar gas constant, approximately equal to 8.314 J·mol^−1^·K^−1^.

(2) $$\ln k=\ln A-E_{a}/RT$$

**Figure 9:**
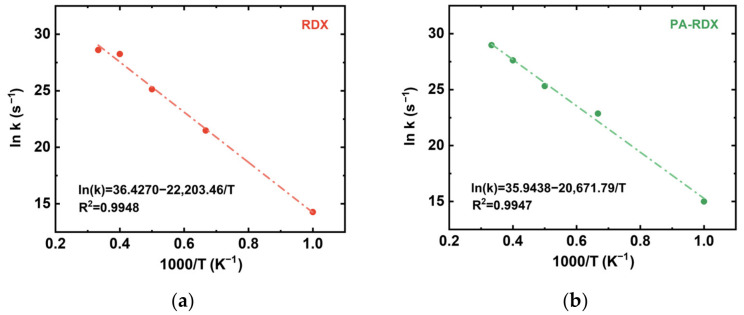
The logarithm of the fitting rate constant ln *k* and the reciprocal of temperature 1/*T*, according to the decomposition simulation results of the system: (**a**) RDX; (**b**) PA-RDX.

The rate constant *k* was calculated by multiple simulations, and the average value at each temperature was taken to fit the Arrhenius expression, as shown in Figure 9.

The activation energies calculated from ReaxFF MD simulation and DSC experimental data in the literature [10], along with their errors, are listed in Table 1. As shown in the table, the activation energies obtained from the simulations are in good agreement with the DSC experimental results, and all relative errors are less than 10%. This indicates the good accuracy of the simulation results and further validates the robustness and reliability of the ReaxFF MD study.

### 2.2. DFT Calculations Analysis

To quantify the interaction strength between PA and RDX, the binding energy of the PA-RDX complex was calculated. The binding energy is defined as $\Delta E=E_{\mathrm{PA-RDX}}-(E_{\mathrm{PA}}+E_{\mathrm{RDX}})$. The calculation results indicate that the total binding energy between PA and RDX is −15.59 kcal/mol, which falls within the typical physical adsorption energy range, suggesting that no covalent bond has been formed between them. The dispersion correction contributes −13.56 kcal/mol, indicating that van der Waals interaction is the main driving force for PA adsorption on the RDX surface. The remaining binding energy (approximately −2.03 kcal/mol) originates from the weak electrostatic attraction between amide groups and nitro groups, as well as the electron density polarization effect. This suggests that there is a significant weak interaction between PA and RDX, rather than a simple electrostatic or orbital effect.

**Figure 10:**
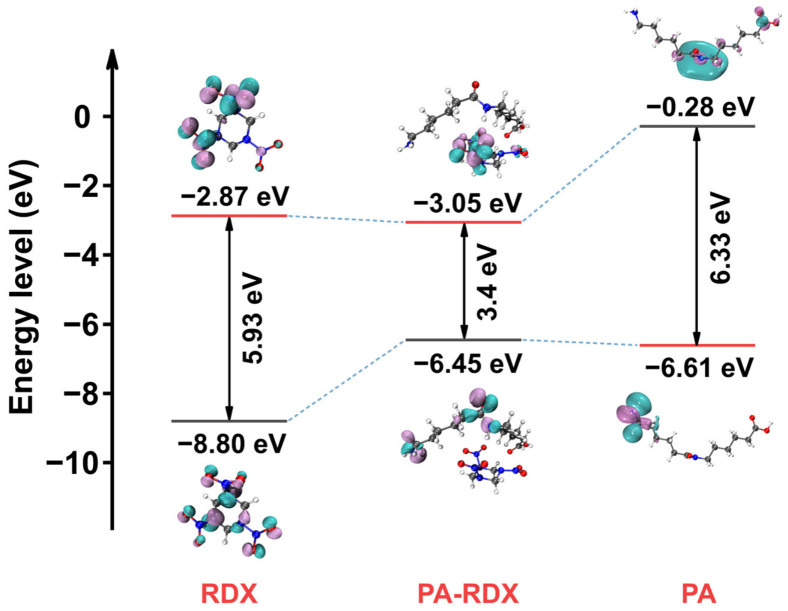
HOMO-LUMO surfaces and energy values of RDX and PA-RDX.

**Figure 11:**
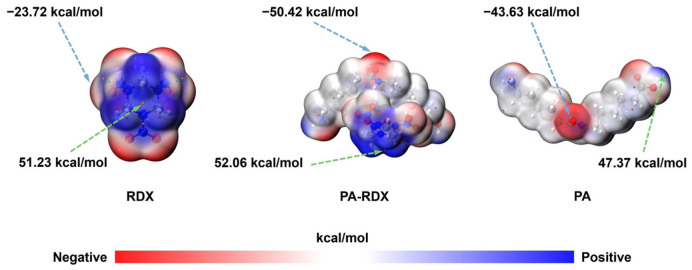
Molecular electrostatic potential of the optimized RDX and PA-RDX.

Based on the characterization results in Figure 10 (HOMO-LUMO energy levels) and Figure 11 (molecular electrostatic potential, ESP), the mechanism by which PA promotes the thermal decomposition of RDX is explained from the dual dimensions of electronic structure and charge distribution.

The HOMO-LUMO energy level diagram in Figure 10 shows that the HOMO energy level of RDX is −8.80 eV and the LUMO energy level is −2.87 eV, resulting in a HOMO-LUMO energy gap (electronic transition energy barrier) of 5.93 eV. The HOMO energy level of the isolated PA fragment is −6.61 eV, and the LUMO energy level is −0.28 eV. After the introduction of PA to form the PA-RDX complex, the HOMO energy level shifts up to −6.45 eV (close to the HOMO of isolated PA), and the LUMO energy level is slightly adjusted to −3.05 eV, with the energy gap significantly reduced to 3.4 eV. The significant decrease in the energy gap of the complex is mainly attributed to the higher HOMO energy level of PA itself, indicating that PA acts as the main source of electrons. At the same time, the weak perturbation of PA on RDX also slightly shifts the LUMO downwards. According to the frontier orbital theory, the HOMO-LUMO energy gap is a core indicator of molecular electronic activity. The decrease in the energy gap means that the introduction of PA reduces the transition energy barrier from occupied orbitals to unoccupied orbitals, enhancing the electronic activity of the system and providing more favorable conditions for electron transfer during thermal decomposition.

Based on the ESP analysis in Figure 11, the electrostatic energy intuitively reflects the charge distribution on the molecular surface and serves as a key basis for identifying reactive sites. The maximum and minimum values of the surface electrostatic potential of RDX are 51.23 kcal/mol and −23.72 kcal/mol, respectively. Relatively speaking, the overall lack of electrons (blue part) and poor electron-rich ability (red part) lead to a scarcity of reactive active centers, which is not conducive to the initiation of the initial step of thermal decomposition. As a comparison, the maximum value of the surface electrostatic potential of an isolated PA molecule itself is 47.37 kcal/mol, and the minimum value is as low as −43.63 kcal/mol, indicating that PA has significant electron-rich ability (strong negative potential region) when it exists alone. However, when PA forms a complex with RDX (PA-RDX), the heterogeneity in the surface electrostatic potential is further enhanced: a more pronounced negative potential enrichment region appears in the PA region (minimum value reaches −50.42 kcal/mol), which is further reduced compared to the −43.63 kcal/mol of isolated PA; meanwhile, the positive potential region becomes more dispersed on RDX, and the maximum value slightly increases to 52.06 kcal/mol (isolated RDX is 51.23 kcal/mol). It is worth noting that the negative potential of the PA part in PA-RDX is stronger than that of isolated PA, while the positive potential of the RDX part is slightly higher than that of isolated RDX, clearly indicating a weak charge/electron transfer from PA to RDX. Therefore, the PA-RDX complex constructs a pair of heteroactive sites on the molecular surface, namely, “electron enrichment (PA side)–electron deficiency (RDX side)”, creating highly active centers for subsequent thermal decomposition reactions.

This heterogeneous distribution of electron-rich and electron-deficient sites creates conditions for electrostatic polarization of the RDX electron density by the PA amide group. This polarization redistributes the electron cloud around the N–NO_2_ bond, drawing density toward the nitro group and thereby weakening the N–N bond, thereby establishing more highly reactive centers on the molecular surface.

Regarding the thermal decomposition of RDX, its initiation process is typically directly related to the homolysis of N–N bonds. After the introduction of PA, the negatively charged regions enriched with electrostatic potential on the molecular surface induce polarization of the electron cloud around adjacent N–N bonds, thereby weakening their bonding energy. Simultaneously, the enhanced electronic activity resulting from the narrowed HOMO-LUMO energy gap, combined with the increased quantity of active sites due to electrostatic potential heterogeneity, collectively lowers the reaction energy barrier for the initial steps of thermal decomposition. In summary, the characterization results of HOMO-LUMO energy levels and molecular electrostatic potential demonstrate, from two core dimensions—electronic activity and charge distribution—that the introduction of PA can effectively promote the thermal decomposition process of RDX.

**Figure 12:**
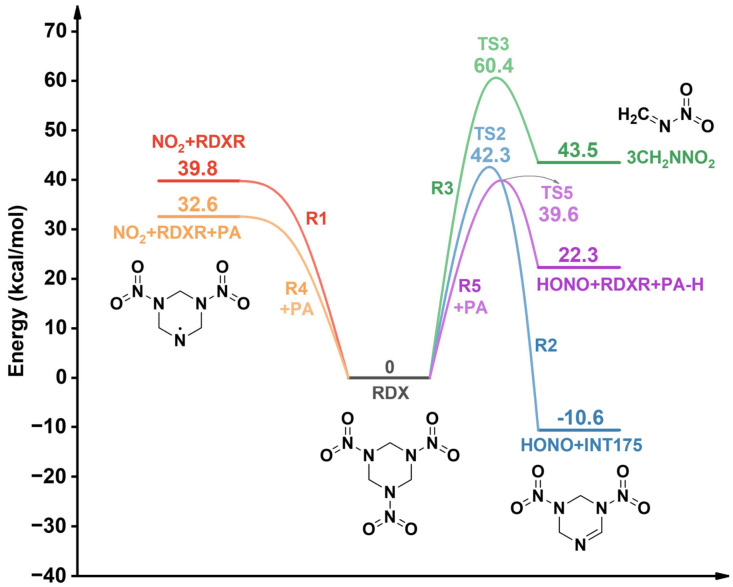
Potential energy profile for unimolecular and bimolecular decomposition of RDX.

The initial decomposition of RDX can proceed through multiple reaction pathways. In this study, we primarily consider three initial decomposition pathways for a single RDX molecule [46]: homolysis of N–NO_2_ to generate RDXR (C_3_H_6_N_5_O_4_) and NO_2_ (R1), HONO elimination to produce INT175 (C_3_H_5_N_5_O_4_) (R2), and a concerted ring-opening reaction yielding three CH_2_NNO_2_ radicals (R3). Additionally, since the introduction of PA leads to increased generation of NO_2_ and HONO during RDX decomposition, PA-assisted reactions for the formation of these two small molecules (R4, R5) are also proposed to explain its promoting effect on RDX decomposition. The calculated energy results for the initial decomposition reactions of RDX are shown in Figure 12.

For the N–NO_2_ homolysis reaction (R1), the result calculated in this work at the B3LYP/6-311+G(d,p) level is 39.8 kcal/mol, which is close to the value of 40.9 kcal/mol reported by Zhang et al. [46] and slightly higher than the 39.0 kcal/mol calculated by Chakraborty et al. [43] at the B3LYP/6-31G(d) level. The transition state energy barrier for the HONO elimination reaction (R2) was reported as 40.2 kcal/mol by Zhang et al. [46] and as 39.2 kcal/mol in the work of Chakraborty et al. [43], both lower than the value of 42.3 kcal/mol (TS2) obtained in this study. In contrast, the barrier of 45.3 kcal/mol calculated by Chen et al. [57] at the M06-2X/jun-cc-pVTZ level is higher than our computational result. For reaction R3, the activation energy barrier for the formation of three CH_2_NNO_2_ radicals is as high as 60.4 kcal/mol, which is close to the 59.3 kcal/mol reported by Zhang et al. [46] and the 59.4 kcal/mol calculated by Chakraborty et al. [43]. Among the three initial reaction pathways, the energy barrier for reaction R3 ranks the highest. Such a high-barrier reaction is less likely to occur compared to the other two initial reactions and therefore will not be discussed in detail.

Calculations indicate that for the decomposition of RDX after the addition of PA, the energy barrier for the homolysis of the N–NO_2_ bond (R4) is 32.6 kcal/mol, which is lower than that of reaction R1 in pure RDX. It should be noted that the decrease in the energy barrier of R4 relative to R1 reflects the catalytic effect of PA on the decomposition of RDX. Although this article did not calculate the control system of the RDX-RDX dimer, the unique hydrogen bond donor/acceptor properties of PA amide groups are fundamentally different from the weak dipole interaction of RDX, making PA chemically specific in stabilizing the transition state of N–NO_2_ bond breakage. ESP analysis reveals that the oxygen in the amide bond (C=O) and the nitrogen (N–H) in PA act as electron donors, enabling weak charge transfer with the electron-deficient nitrogen atom in the NO_2_ group of RDX. This directly weakens the N–NO_2_ bond in RDX and reduces the energy required for its homolysis. The energy barrier (TS5) for the reaction (R5) in which RDX extracts H from PA to form HONO is also 2.7 kcal/mol lower than that of the HONO elimination reaction (R2) in pure RDX. The addition of PA reduces the energy barriers for both initial decomposition pathways of RDX, indicating that PA makes early-stage decomposition of RDX more favorable, which is consistent with the results discussed in Section 2.1.2.

### 2.3. Decomposition Reaction Mechanism

In the previous two sections, we analyzed the product evolution and primary reaction pathways of RDX during thermal decomposition, as well as the impact of PA addition on this process, by triplet experiments, ReaxFF MD simulations and DFT calculations. In this section, we will provide a further elaboration on the reaction mechanisms of the two systems.

**Figure 13:**
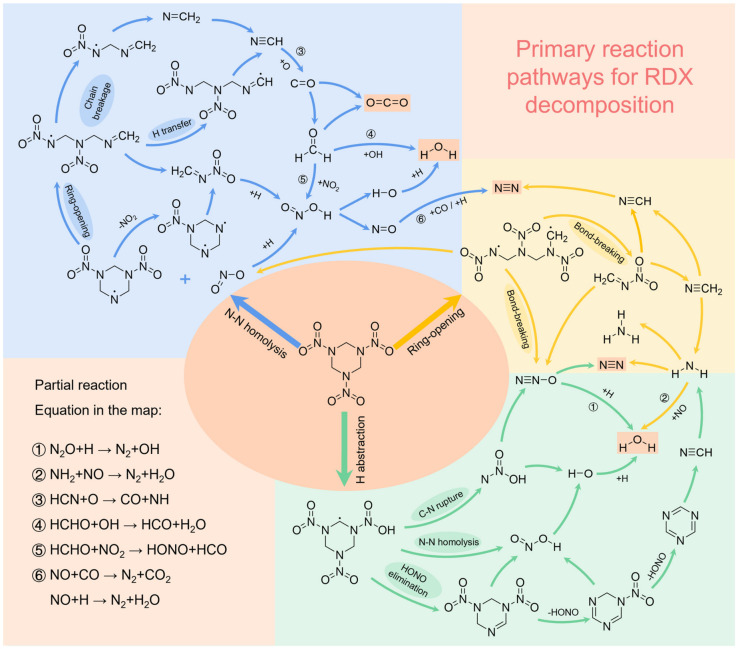
The main reaction paths of the RDX thermal decomposition process. (The blue region represents N–N bond homolysis and the subsequent chain reaction; the yellow region represents the chain reaction after direct ring-opening fracture; the green region represents a series of chain reactions after H abstraction).

Figure 13 illustrates the main reaction pathways during the thermal decomposition of RDX. The central oval section in the diagram represents the initial decomposition steps of RDX, which serve as the starting point for the entire decomposition process. Typically, there are three pathways: (1) Homolysis of the N–NO_2_ bond: This generates the crucial primary radical NO_2_, which can then attack adjacent RDX molecules or residual organic fragments to abstract H atoms, thereby initiating chain reactions. This pathway is the dominant route for RDX decomposition (through the analysis in Section 2.1.2, we also concluded that N–N bond homolysis is the first step in RDX decomposition). (2) H abstraction reaction: Reactive H atoms are directly abstracted by the –NO_2_ groups on RDX molecules, subsequently triggering a series of bond-breaking and chain reactions. (3) Concerted ring-opening homolysis: The RDX molecule undergoes ring-opening, followed by concerted cleavage of C–N bonds, generating three identical radicals, which then further decompose to form products such as NO_2_, N_2_O, and H_2_CN.

The high-temperature decomposition of RDX is an extremely rapid and complex free-radical chain reaction process. Its core characteristics include multi-path initial decomposition and rapid oxidation equilibrium. The reaction begins with bond homolysis and ring-opening of RDX molecules, instantly producing key intermediates such as NO_2_, NO, HONO, HCN, and HCHO, which act as “seeds” for subsequent reactions. These intermediates rapidly dissociate, forming a highly reactive “reaction pool” consisting of radicals such as H·, O·, ·OH, N·, NO·, and ·CN, marking entry into the core reaction stage. During this stage, various elements undergo complex interactions and transformations. Nitrogen participates in intense self-redox reactions, tending to form the most stable product N_2_ through radical recombination, while also generating minor amounts of by-products such as N_2_O and NO. Carbon is vigorously oxidized by oxygen radicals (O·, ·OH), with a key pathway being the oxidation of CO by ·OH to form CO_2_. Species such as HCN and HCHO serve as important precursors to CO. Hydrogen, in the form of H· radicals, acts both as a reducing agent—facilitating the conversion of NO_2_ and NO into N_2_—and as a key reactant that combines with oxygen radicals to form H_2_O. The entire reaction network is essentially a fierce competition, with oxygen acting as the oxidizer against carbon, hydrogen, and some nitrogen-containing intermediates. Driven by thermodynamic equilibrium, the reaction strongly favors the formation of the most stable molecules: N_2_, H_2_O, and CO_2_. Although some final products (e.g., H_2_O, CO_2_) may undergo reversible reactions (e.g., CO_2_ + H → CO +·OH), the overall process exhibits a strong tendency toward generating N_2_, H_2_O, and CO_2_.

**Figure 14:**
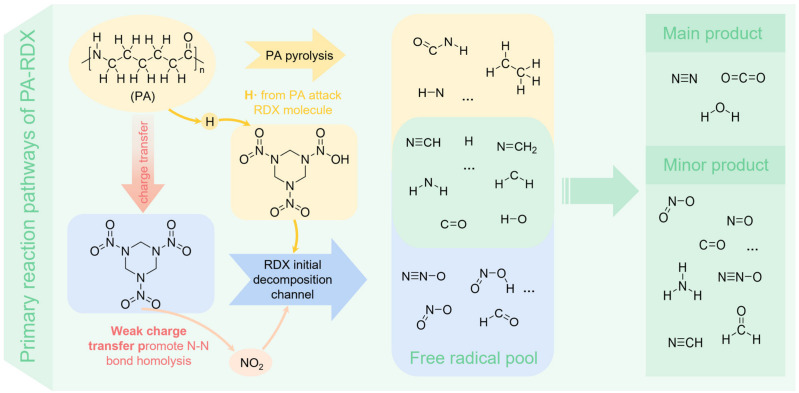
Thermal decomposition reaction mechanism of PA-RDX. (The yellow area represents part of the intermediate products of PA thermal decomposition; the blue region represents part of the intermediate products of RDX thermal decomposition; the intermediate green area is the common intermediate product of the thermal decomposition of PA and RDX. The rightmost green area represents the final product of the whole system).

The interaction between PA and RDX also exhibits characteristics of a free radical chain reaction, as shown in Figure 14. After the reaction begins, a weak charge transfer occurs from the electron-rich regions on the PA molecular chain to the electron-deficient regions on RDX, slightly lowering the energy barrier for N–NO_2_ homolysis and promoting the initiation of RDX decomposition. The active H atoms generated from PA pyrolysis directly attack RDX molecules, triggering HONO elimination reactions. This heterogeneous intermolecular attack is more efficient than H-abstraction within RDX itself, further accelerating the decomposition process. The PA molecular chain breaks apart, primarily producing H·, ·CO, HCN, ·NH_2_, HCHO, and small hydrocarbon molecules. Meanwhile, RDX decomposes to generate its characteristic products along with a large number of reactive radicals, such as O· and ·OH. These primary fragment products together form a highly reactive radical pool and a key intermediate reaction system, which then enters an intense stage of radical reactions.

The fragments from both substances undergo deep cross-interactions: Oxidizing radicals (O·, ·OH, ·NO_2_) provided by RDX oxidize the hydrocarbon fragments derived from PA, gradually converting them into CO and ultimately oxidizing them to CO_2_. Simultaneously, the ·NH_2_ radicals supplied by PA undergo a crucial reaction with ·NO generated from RDX (via the ·NH_2_ +·NO → N_2_ + H_2_O pathway), efficiently producing stable N_2_ and H_2_O. HCN, as an important common intermediate, is oxidized by ·OH, O·, etc., contributing to the formation of N_2_, N_2_O, and NO through pathways involving HNCO and NCO. N_2_O is mainly consumed through thermal decomposition and reduction by hydrogen atoms. Ultimately, the reaction tends to produce the thermodynamically most stable products: N_2_, CO_2_, and H_2_O. However, due to high-temperature kinetic control and the initial product distribution, the system still contains certain amounts of minor products, such as CO, N_2_O, NO, NO_2_, HCN, HCHO, and NH_3_, which are direct reflections of the competing pathways within the reaction network.

The traditional homogeneous decomposition of RDX initiates with the homolysis of the N–NO_2_ bond. The introduction of PA lowers the energy barrier for this reaction, facilitating earlier decomposition. The cleavage of the amide bonds (–CO–NH–) in the PA polymer chain generates a series of small nitrogen- and oxygen-containing fragments, as well as reactive species such as aldehydes and ketones. These can rapidly react with intermediates produced during RDX decomposition, consuming them and thereby shifting the equilibrium of RDX decomposition toward the forward reaction direction. As a hydrogen-rich and carbon-rich polymer, PA provides abundant fuel—such as CO and hydrocarbon fragments generated from the cleavage of its long carbon chains and methylene structures—for the strongly oxidizing radicals derived from RDX. This enhances the overall oxidation extent of the system, resulting in the formation of more CO_2_ and H_2_O.

In summary, the reactive radicals provided by PA participate in chain reactions, serving as both initiators and propagators. By offering heterogeneous initiation pathways with lower energy barriers, PA accelerates the entire decomposition process, effectively replacing the higher-energy self-initiation process required by RDX. This significantly promotes the decomposition of RDX at the chemical reaction level.

## 3. Discussion

Based on previous research, we found that PA promotes the thermal decomposition of RDX. The 3000 K simulation conditions set in molecular dynamics simulations can be approximately considered as instantaneously igniting the system, resembling the process of an explosive being detonated upon impact. Next, we extrapolate the theoretical findings to the process of RDX explosive initiation under jet impact.

Under shock initiation conditions, the decomposition of RDX manifests as an extremely rapid and highly complex free-radical chain reaction. The core mechanism involves multi-path decomposition and a dynamic balance of rapid oxidation triggered by the high-temperature and high-pressure environment instantaneously generated under shock wave loading. During the formation of hot spots under impact loading, RDX molecules primarily undergo rapid decomposition through bond dissociation and concerted ring-opening, producing HCN and NO_2_ as the dominant primary intermediates while generating components such as HCHO, NO, and HONO. These highly reactive intermediates immediately undergo further decomposition under high-temperature conditions: NO_2_ undergoes homolysis to produce NO and reactive O·, while HCN rapidly reacts with radicals such as O· and ·OH, eventually oxidizing to CO and N_2_ via intermediate forms like NCO. HCHO undergoes dehydrogenation to form HCO, which is further converted to CO. During this process, the abundant production of O·, H·, ·OH, and N· radicals, constituting the core carriers of chain reactions, drives intense secondary oxidation and decomposition reactions. The entire system rapidly approaches thermodynamic equilibrium through efficient radical recombination and oxidation pathways. Ultimately, nitrogen is primarily converted to stable N_2_, hydrogen combines with oxygen radicals to form H_2_O, and carbon is mainly transformed into CO_2_. Therefore, the primary products after RDX explosion are stable substances, such as N_2_, H_2_O, and CO_2_, along with minor products in lower yields. Due to their high reactivity, intermediates such as HCN, HCHO, and CO exhibit extremely low concentrations at equilibrium (typically <1%). This reaction completes on a microsecond or even shorter timescale, reflecting the ultra-fast energy release characteristic of impact-induced initiation.

During the process of PA jet impact on RDX explosives, the introduction of PA provides a lower-energy-barrier heterogeneous decomposition pathway for the system, significantly promoting the rapid initiation of RDX. This mechanism bypasses the slow homogeneous ring-opening initiation step intrinsic to RDX itself, primarily realized through the following processes: PA undergoes rapid pyrolysis under impact, with cleavage of amide bonds (–NH–CO–) and C–C bonds, generating active radicals such as H· and ·OH, as well as small-molecule compounds including aldehydes, ketones, and nitrogen-containing organic species. Among these, H· radicals attack RDX molecules, facilitating the homolytic cleavage of N–N bonds and releasing products such as HONO. The decomposition of HONO produces ·NO and ·OH, which sharply trigger an explosive chain reaction, establishing an autocatalytic cycle. The entire process lowers the activation energy barrier and accelerates the propagation and expansion of the radical chain, thereby enabling rapid reaction initiation. Ultimately, this ensures the initiation of RDX under the impact of a PA jet.

It should be noted that the ReaxFF MD simulation in this paper employs a uniform heating approach, aiming to reveal the intrinsic chemical reaction pathways and kinetic characteristics of the PA-RDX interface at high temperatures, rather than simulating shock wave propagation or macroscopic hotspot expansion processes. The simulation results provide mechanistic chemical insights into experimental observations. The mechanistic explanation proposed in this study, through theoretical calculations, provides a preliminary physicochemical understanding of the PA jet initiation of RDX. Limited by the inherent scale of reactive molecular dynamics simulations and the uniform heating strategy adopted in this paper, this study cannot directly describe the macroscopic physical processes, such as hot spot formation, non-uniform heat transfer and long-range material diffusion, involved in the impact initiation process. Therefore, the effect of PA on the thermal decomposition pathway of RDX revealed in this paper should be understood as a local chemical interaction mechanism at the interface between PA and RDX at high temperature, rather than a full-scale physical reproduction of the impact-induced detonation phenomenon. Nevertheless, these mechanistic understandings at the molecular level provide a direct chemical basis for explaining the promotion effect of PA on the thermal decomposition of RDX observed in the experiment. Therefore, future work will focus on dynamic loading conditions that more closely resemble real initiation scenarios by conducting molecular/atomic-scale simulations that integrate mechanical, thermal, and chemical coupling. Within a multiscale framework, this approach will directly link material response to initiation mechanisms, thereby establishing a more precise physicochemical model.

## 4. Materials and Methods

### 4.1. Reaction Molecular Dynamics Simulation Method

#### 4.1.1. ReaxFF Molecular Dynamics

ReaxFF MD enables the investigation of complex chemical reactions in large-scale systems. It employs a bond-order formalism combined with polarizable charges to describe both reactive and non-reactive interatomic interactions, allowing accurate simulation of covalent and electrostatic interactions in a wide range of materials [58].

In ReaxFF, the connectivity between any two atoms is dynamically determined, thereby facilitating the representation of bond breakage and formation. The total energy of the system is expressed as:

(3) $$E_{system}=E_{bond}+E_{over}+E_{under}+E_{val}+E_{pen}+E_{tors}+E_{conj}+E_{vdWaals}+E_{Coulomb}$$

where *E_system_* is the total energy of the system; *E_bond_* represents bond energy, reflecting the strength of covalent bonding between atoms; *E_over_* and *E_under_* denote over-coordination and under-coordination energy penalties, respectively; *E_val_* represents valence angle energy, which maintains molecular structural stability; *E_pen_* is a penalty energy term that corrects stability issues in valence angles not fully captured by *E_val_*, particularly in systems with atoms involved in multiple double bonds; *E_tors_* represents torsional angle energy; *E_conj_* is the energy contribution from conjugation effects; *E_vdWaals_* describes nonbonded van der Waals interactions; and *E_coulomb_* represents Coulombic electrostatic interactions. During simulations, the bond orders between atoms are continuously updated based on evolving atomic positions [59].

Potential energy can be divided into bond-order-dependent and bond-order-independent contributions. The bond order is directly computed from the interatomic distance updated at each time step:

(4) $$BO_{ij}=BO_{ij}^{\sigma }+BO_{ij}^{\pi }+BO_{ij}^{\pi \pi }=\exp\left[p_{bo1}{\left(\frac{r_{ij}}{r_{0}^{\sigma }}\right)}^{p_{bo2}}\right]+\exp\left[p_{bo3}{\left(\frac{r_{ij}}{r_{0}^{\pi }}\right)}^{p_{bo4}}\right]+\exp\left[p_{bo5}{\left(\frac{r_{ij}}{r_{0}^{\pi \pi }}\right)}^{p_{bo6}}\right]$$

where *BO_ij_* represents the bond order between atoms *i* and *j*, *r_ij_* is the interatomic distance, *r_o_* terms denote equilibrium bond lengths, and *p_bo_* terms are empirical parameters. This formulation ensures continuity in transitions among *σ*, *π*, and *ππ* bond characteristics, resulting in a differentiable potential energy surface suitable for calculating interatomic forces. The bond-order expression accounts for long-range covalent interactions in transition-state structures, enabling the force field to accurately predict reaction barriers. It is important to note that in ReaxFF, nonbonded and bonded interactions are computed independently—there is no information exchange between bond-order-dependent terms and van der Waals or Coulomb-related terms. Bond-order-based terms and nonbonded terms are evaluated for all materials and molecules [60].

#### 4.1.2. Model Construction

**Figure 15:**
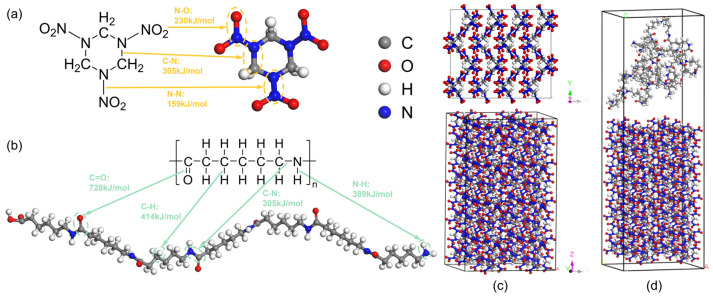
Molecular structure and system configuration of RDX and PA-RDX: (**a**) molecular structure of RDX; (**b**) molecular structure of PA; (**c**) initial configuration of RDX system; (**d**) initial configuration of PA-RDX system.

The focus of this study is the reaction kinetics of the explosive molecule (RDX) and the composite system composed of the polymer molecule (PA) and RDX. The chemical formulas and molecular structures of the two molecules are shown in Figure 15a,b. In the chemical formulas, “n” represents the degree of polymerization of the polymer chain; for PA, this value was set to 7 [61].

The structural models of RDX molecules and the composite of PA and RDX molecules were constructed in two periodic cubic boxes by Materials Studio 23.1 (MS) software. The initial RDX crystal structure was obtained from the Cambridge Crystallographic Data Centre (CCDC, CCDC number 1131953) [62]. This structure corresponds to the thermodynamically stable *α*-RDX phase, belonging to the orthorhombic crystal system with space group *Pbca*. The unit cell parameters are *a* = 13.182 Å, *b* = 11.574 Å, and *c* = 10.709 Å, containing 8 RDX molecules per cell. The initial RDX unit cell was expanded into a 2 × 2 × 4 supercell. Subsequently, a composite system model was built via interfacial structure formation, combining this supercell with PA molecules at a content ratio of 9:1 (RDX:PA). The RDX crystal structure after supercelling was cut along the (001) crystal plane, retaining an RDX surface slab layer with a thickness of approximately 10 Å. A vacuum layer with a thickness of 20 Å was added above the RDX slab layer to eliminate false interactions caused by periodic boundary conditions in the z direction. The Amorphous Cell module was used to construct an amorphous PA structure, which was placed within the vacuum layer region, and its initial position was controlled to be approximately 3.0 Å away from the RDX surface to avoid initial overlap between atoms. The resulting model structures are depicted in Figure 15c,d. The atom counts for the pure RDX and PA-RDX systems are 2688 and 3232, respectively, with corresponding mass densities of 1.82 g/cm^3^ and 1.12 g/cm^3^. To ensure system stability, both models underwent geometry optimization at 300 K to obtain their minimum energy structures.

Molecular dynamics simulations were performed on a Large-scale Atomic/Molecular Massively Parallel Simulator (LAMMPS, LAMMPS-64bit-29Aug2024-MSPI), developed by Sandia National Laboratories in the United States. The ReaxFF-lg force field for C/H/O/N developed by Lianchi Liu et al. was employed, which has been widely applied in reactive molecular dynamics simulations of energetic materials, including RDX and its polymer-modified systems [63]. The ReaxFF-lg force field was originally parameterized and validated against graphite and polyethylene (PE) to accurately describe the London dispersion interactions in organic and polymeric systems. The force field includes parameters for C, H, O, and N elements and has been successfully applied to study the thermal decomposition of other polymers such as epoxy resins and polyimide. Therefore, the applicability of ReaxFF-lg to PA systems is supported by its prior validation on polyethylene and other polymers containing the same constituent elements. The Velocity Verlet algorithm was selected to simulate the high-temperature reaction processes of each system based on the ReaxFF MD methodology.

The conjugate gradient method was used to minimize the energy of the initial configuration to eliminate unreasonable atomic contact and excessive internal stress in the system. The double convergence criterion was set in the optimization process: the change value of the total energy of the system was ≤1.0 × 10^−4^ kcal/mol, and the maximum force of the atom was ≤0.005 kcal·mol^−1^·Å^−1^. Under the NVT ensemble, the system that completed the geometric optimization was annealed at room temperature. A Nosé–Hoover hot bath was used to control the temperature. The damping constant of the hot bath was set to 100 fs, and the temperature of the system was maintained at 300 K. The total simulation time was 100 ps, and the integral time step was set to 0.25 fs. Through this equilibrium process, the system reaches a thermodynamically stable state at room temperature, which provides a reliable initial equilibrium configuration for subsequent decomposition reaction kinetics simulations. Subsequently, thermal decomposition simulations were conducted. A temperature of 3000 K was adopted to represent instantaneous ignition of the systems, approximating the detonation process of explosives under shock loading. Hence, the equilibrated structures of each system were subjected to high-temperature decomposition simulations under the NVT ensemble at 3000 K, with a timestep of 0.1 fs and a total simulation time of 300 ps. In all simulations, the temperature of each system was regulated using a Berendsen thermostat [64] to maintain fluctuations around the set value.

### 4.2. DFT Calculation

All DFT calculations were performed using Gaussian 16. The structures of various species obtained from ReaxFF MD simulations—including reactants, intermediates (IMs), transition states (TSs), and products—were optimized at the B3LYP/6-311+G(d, p) level with Grimme’s D3 dispersion correction [46]. For open-shell species, unrestricted Kohn–Sham equations were employed according to the corresponding spin multiplicity. To confirm that each optimized structure corresponds to a local minimum or a saddle point, harmonic frequency calculations were carried out at the same level of theory. The transition state was identified by the presence of exactly one imaginary frequency. For ambiguous transition states, intrinsic reaction coordinate (IRC) calculations were performed to verify that the identified TS correctly connects the intended reactants and products [65]. Reaction mechanisms for all pathways were determined based on IRC analysis. Zero-point energy (ZPE) corrections were applied to all energy results reported in this work.

**Figure 16:**
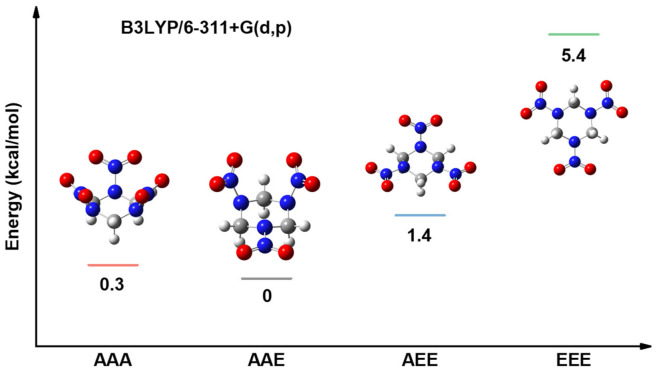
Relative energy of RDX conformations.

Figure 16 displays the four optimized stable configurations of RDX and their relative energies. These structures are named according to the positions of the nitro groups, designated as axial (A) and equatorial (E), and are labeled as AAA, AAE, AEE, and EEE. The relative energy calculations show that the AAE configuration—with one equatorial nitro group and two axial nitro groups—is the most stable RDX conformation, which is consistent with previous studies [46,47]. Therefore, the AAE configuration of RDX was selected for subsequent computational investigations. In addition, a composite model of PA and RDX molecules was constructed, and the electrostatic potential and HOMO/LUMO surfaces of the composite structure were determined to analyze the influence of PA on the decomposition reaction of RDX. The initial configuration of PA-RDX was derived from the last 5 ps of the relaxation trajectory obtained from ReaxFF MD at 300 K. Snapshots were taken every 1 picosecond, resulting in a total of five PA-RDX configurations. To reduce computational costs, the PA chain was truncated to a fragment containing two amide repeat units, with the cut-off point saturated with hydrogen atoms, while the RDX molecule remained intact. Full geometric optimization was performed on each configuration at the B3LYP/6-311G+(d,p) theoretical level, with D3 dispersion correction applied. Frequency analysis confirmed that all optimized configurations had no imaginary frequencies. The electronic energies of each optimized configuration were compared, and the configuration with the lowest energy and no imaginary frequencies was selected as the input structure for subsequent calculations.

## 5. Conclusions

To elucidate the physicochemical mechanism underlying violent detonation phenomena when a PA polymer jet impacts explosive reactive armor, the thermal decomposition behavior of RDX and the influence mechanism of PA on the decomposition reaction of RDX were systematically studied by ReaxFF MD simulations and DFT calculations. The main conclusions are as follows:

(1) Based on ReaxFF MD calculations, the primary initial reaction pathways for RDX decomposition were determined: homolytic cleavage of the N–NO_2_ bond, HONO elimination, and concerted ring-opening decomposition.
(2) The reactive radicals generated from PA decomposition participate in chain reactions, acting as initiators and propagators. They undergo cross-reactions with oxygen/nitrogen-containing radicals produced during RDX decomposition, collectively yielding small molecular products. Oxidizing radicals derived from RDX progressively oxidize hydrocarbon fragments derived from PA into CO_2_, while ·NH_2_ radicals provided by PA efficiently react with ·NO generated from RDX via a key reaction to produce N_2_ and H_2_O. These synergistic pathways drive the reaction network toward the formation of thermodynamically stable final products and alter the distribution and concentration of the products.
(3) DFT calculations revealed that PA promotes the thermal decomposition of RDX, primarily through a dual mechanism that lowers the initial decomposition energy barriers of RDX: electron-rich regions in PA perturb and weaken the N–NO_2_ bonds of RDX via interfacial charge transfer, reducing their homolysis barrier; meanwhile, active hydrogen atoms generated from PA pyrolysis provide an efficient heterogeneous hydrogen source for RDX, facilitating the occurrence of HONO elimination reactions. These effects make the initial decomposition reactions of RDX easier to initiate.

In this paper, the mechanism by which PA promotes RDX thermal decomposition is explained from the perspective of microscopic reactions, and mechanistic insights into possible reaction pathways are provided. However, the current model seems to represent a highly idealized reaction system rather than a true description of the impact-induced interfacial decomposition process. In the future, the cross-scale method will be used to further bridge the relationship between the molecular mechanism and the behavior of macroscopic energetic materials.

## Figures and Tables

**Table 1:** Comparison of the activation energy calculated by DSC experimental and ReaxFF MD.

	RDX	PA-RDX
DSC experiment [10]	198.14 kJ/mol	180.88 kJ/mol
ReaxFF MD caculation	184.60 kJ/mol	171.87 kJ/mol
Error	6.834%	4.981%

## Data Availability

Data are contained within this article.
